# Editorial: Understanding Age and Sex-Related Differences in the Biomechanics of Road Traffic Associated Injuries Through Population Diversity Analyses

**DOI:** 10.3389/fbioe.2022.869356

**Published:** 2022-05-13

**Authors:** Francisco J. Lopez-Valdes, Sonia Duprey, Jason Forman, Mats Y. Svensson

**Affiliations:** ^1^ Institute for Research in Technology (IIT), ICAI, Universidad Pontificia Comillas, Madrid, Spain; ^2^ Université Claude Bernard Lyon 1, Lyon, France; ^3^ University of Virginia, Charlottesville, VA, United States; ^4^ Chalmers University of Technology, Göteborg, Sweden

**Keywords:** road traffic injuries (RTI), population diversity, impact biomechanics, biofidelity, human body model (HBM)

Road traffic injuries account for 1.35 million deaths and approximately 50 million injuries yearly according to the World Health Organization (WHO, 2019). These injuries are unequally shared by the world’s population, with several vulnerable groups being overexposed to the effects of injuries. For instance, road injuries are the leading cause of death for children and young adults (5–29 years old). Recent research has pointed out that women are at a greater risk of death and of sustaining severe injuries under the same crash configurations as men (Bose et al., 2011). Elderly car occupants have been identified as particularly vulnerable to the deployment of contemporary safety systems such as airbags and seatbelts (Kent et al., 2009).

While the seminal work done on Injury Biomechanics in the 1970’s–1980’s provided data to develop injury criteria that can be used with Anthropometric Test Devices (ATD), also known as crash test dummies or just dummies, there is a growing body of literature pointing out to the need of recognizing how differences between individuals may modify their specific risk to injuries (Forman et al., 2015). The source of this variability is not unique, but more and more research suggests that anthropometry, age and sex are significant factors influencing the injury tolerance of individuals.

Thus, the goal of this Research Topic is to highlight how these biomechanical differences between population groups are identified and eventually incorporated into the design of effective safety systems capable of preventing injuries for all road users.

Biomechanical research always needs to keep the connection with real world injuries. The study “Are There Any Significant Differences in Terms of Age and Sex in Pedestrian and Cyclist Accidents?” analyzes sex-specific differences in pedestrians and cyclists collisions in three European countries finding that women are at higher risk of sustaining AIS3+ lower extremity and pelvic injuries (OR = 2.11–3.03, depending on the country, but statistically significant for all analyzed countries). Two articles looked at how women and men react to longitudinal accelerations in standing position as in public transportation situations. The study “Human Response to Longitudinal Perturbations of Standing Passengers on Public Transportation During Regular Operation” found out shorter muscle response time in female volunteers compared to their male counterparts, while the paper “Identifying and Characterizing Types of Balance Recovery Strategies Among Females and Males to Prevent Injuries in Free-Standing Public Transport Passengers” found no differences between the two sexes in the balance recovery outcome to longitudinal perturbations, although no statistical comparison could be made between different recovery strategies due to the sample size of the volunteer group (*n* = 24). The subject deserves further research as it was found that while seven out of 13 males used the “fighting stance” as the recovery strategy only three out 11 females adopted this strategy to maintain balance.

Two studies used computational modeling to analyze whether existing head injury criteria were suitable to predict traumatic brain injury in the elderly. “The head AIS4+ injury thresholds for the elderly vulnerable road user based on detailed accident reconstructions” reconstructed 30 real world cases to find that currently proposed injury thresholds for traumatic brain injury, both based on linear or angular magnitudes, should be substantially lowered to capture the injury likelihood of the elderly population (for instance, the found threshold of HIC_15_ for AIS4+ injuries in this study was 1,082 compared to the 1,440 threshold proposed by NHTSA). Following a similar methodology, “Evaluation of Head Injury Criteria for Injury Prediction Effectiveness: Computational Reconstruction of Real-World Vulnerable Road User Impact Accidents” identified differences between the predictions of strain-based head injury criteria (maximum principal strain was a better predictor of Diffuse Axonal Injury than cumulative strain damage). In the case of kinematics-based injury criteria, the more traditional injury criteria such as HIC and HIP provided comparably accurate results. Computer modeling was also used in “Rib cortical bone fracture risk as a function of age and rib strain: Update injury prediction using Finite Element Human Body Models” to provide an estimation of the risk of rib fractures using a probabilistic approach and experimental data from 58 individuals spanning 17–99 years old, providing a robust framework to advance the use of human body models in the prevention of road traffic injuries.

The differences with age and sex in the cervical spine was the topic of two of the studies submitted to this article collection. “The lack of sex, age and anthropometric diversity in neck biomechanical data” found out that the neck biomechanical data were biased toward males, younger volunteers and older Post Mortem Human Surrogates in a systematic review of the literature. The study “Comparison of Upper Neck Loading in Young Adult and Elderly Volunteers During Low Speed Frontal Impacts” is the first one in the literature comparing the experimentally measured cervical loading of younger (*n* = 9) and older (*n* = 4) volunteers under the same dynamic conditions to find out that there were not substantial differences between the two age groups. This finding was also supported by the study “Sex, Age, and Stature Affects Neck Biomechanical Responses in Frontal and Rear Impacts Assessed Using Finite Element Head and Neck Models” that did not identify overall kinematic differences between an aged cervical model and a younger one, which would justify that the inverse kinematics method used in the previous study could not identify important differences in the cervical loading. In parallel, the study found that aged models predicted higher ligament deformations. The female model also exhibited larger shear forces at the facet joints, agreeing with available epidemiological data. Further research on this area is needed to increase the sample size of the available experimental data and the statistical power of the comparisons between cervical loads across age groups and sexes.

Additional insight into the response of women to rear impacts was provided by three studies. “Dynamic Responses of Female Volunteers in Rear Impact Sled Tests at Two Head Restraint Systems” supplies new experimental data from female volunteers at different dynamic conditions that can be used to develop more biofidelic physical and computational female surrogates. The need for developing female models is also supported by the findings of “The effect of seat back inclination on spinal alignment in automotive seating postures” that identified differences in the lordosis and kyphosis between females and males depending on the seat back inclination in a set of 23 volunteers. The first attempt to develop a physical dummy to represent female occupants in rear impacts is included in “Design and Evaluation of the Initial 50^th^ Percentile Female Prototype Rear Impact Dummy, BioRID P50F- Indications for the need of an additional dummy size”, which presented promising results about the performance of a new more women-like physical model in rear impacts.

In summary, the current Research Topic offers insights into the use of computer models to investigate the performance of existing and newly proposed injury criteria capable of capturing individual differences related to age and sex variations. It also provides new experimental data that can be used in the development of more accurate physical and computational surrogates. And, finally, the collection includes information about the development of a new physical crash test dummy intended to improve the protection of female occupants in rear impacts.

We trust that the amount of new data and developments included in this Research Topic provides valuable information to the field and, above all, contributes to reduce the number of motor vehicle related injuries that can be related to sex and age differences between road users.
